# Long-term potentiation-induced changes in actin dynamics and spine geometry persist on the timescale of the synaptic tag

**DOI:** 10.1038/s42003-025-08459-0

**Published:** 2025-07-18

**Authors:** Mitha Thomas, Cristian-Alexandru Bogaciu, Silvio O. Rizzoli, Michael Fauth

**Affiliations:** 1 Third Institute for Physics, Georg-August University, Friedrich Hund Platz 1, Göttingen, Germany; 2 Department of Neuro- and Sensory Physiology, University Medical Center, Humboldtallee 23, Göttingen, Germany

**Keywords:** Long-term potentiation, Consolidation, Computational biophysics

## Abstract

According to the tagging and capture hypothesis, long-lasting long-term potentiation (LTP) requires protein synthesis and a synaptic tag, which is a synapse specific memory of the stimulus with a so far unclear molecular or biophysical identity. Here we use an interdisciplinary approach to explore the hypothesis that interaction between the dynamics of actin and the spine geometry can provide such a memory. Using a mathematical model, we demonstrate that this implementation of the tag requires an increase in the stable, cross-linked pool of actin filaments, and is not possible without this stable pool. Using FRAP experiments, we show that such an increase in stable actin can be observed hours after chemical LTP induction in vitro. Thus, the interaction between actin dynamics and spine geometry could indeed serve as a synaptic tag for LTP.

## Abstract (web summary)

A mathematical model of dendritic spine membrane interacting with dynamic and stable actin exhibits LTP-induced alterations on the timescale of the synaptic tag due to up-regulation of stable actin which are also observed in FRAP experiments in vitro.

## Introduction

Long-term potentiation (LTP,^1^) of excitatory synapses changes the connectivity of neuronal networks and their ability to process information. Accordingly, LTP has been associated to learning and long-term memory formation in neuronal networks^2–4^. However, long-term potentiation can be expressed in different forms^5,6^: as early LTP (E-LTP), which decays within hours, and as late LTP (L-LTP), which persists much longer. The most common model for the emergence of L-LTP—the synaptic tagging-and-capture hypothesis^5,7^—states that L-LTP requires two components: (1) a transient memory that the synapse experienced a plasticity inducing event, the so-called synaptic tag, and (2) de novo synthesis of plasticity-related proteins (PRPs), which are then translocated to the tagged synapse and give rise to L-LTP. If PRPs are missing, only E-LTP is observed.

The molecular or biophysical identity of the synaptic tag is not completely clear^7,8^, but one molecule that is intimately related to the synaptic tag is the scaffolding protein actin^9^. Actin forms dynamic filaments that continuously polymerize at their barbed end and depolymerize at their pointed end (treadmilling). Additionally, various actin binding proteins (ABPs) organize the filaments into branched networks (ARP2/3 complex), sever the filaments (ADF/cofilin), or cap the barbed ends and prevent polymerization (capping protein)^10^. Moreover, the filaments can be distinguished into (at least) two dynamically distinct pools of actin: a fast-treadmilling, dynamic pool and a stable pool, in which filaments are bound to cross-linkers like *α*-actinin, drebrin, cortactin^11^ or CaMKII^12^ as well as other proteins^13^, which slow down the filament dynamics^14^. These pools undergo massive reorganization during LTP, as the concentration of many actin-binding proteins and cross-linkers changes in a time-dependent manner^15,16^ and their activity is regulated by calcium-dependent signaling. Yet, such transient changes in actin alone cannot account for the tag^7,17,18^.

However, as actin forms the scaffold of the spine, its time-dependent reorganization also gives rise to massive changes in spine geometry—most prominently an enlargement of the spine volume^19^. Thus, there is a complex interaction between actin pools and spine geometry, which may retain the information of a plasticity event longer than actin dynamics alone and would also be consistent with the tag being a “temporary structural state of the synapse"^7^.

To understand this complex interaction, one can use mathematical modeling, which is based on, and informs, experimental measurements. Along this line, a variety of models describing the interaction between actin dynamics and spine geometry have been published^20–25^. Yet, these models usually only consider the dynamic pool of actin filaments that drives the expansion of dendritic spines, and do not explicitly consider the existence or re-organization of the stable pool.

Accordingly, these models can only account for the first few minutes after LTP, where the initial enlargement of the spine takes place. It is, however, unclear whether these models exhibit a persistent change of spine geometry and actin dynamic on the timescale of the synaptic tag, which is typically around one to two hours^5^. Moreover, while there is evidence that cross-linked filaments play a major role during^14^ and after LTP^26^, it is unclear whether the stable pool exhibits any alterations at this timescale.

Therefore, in this study, we follow an interdisciplinary approach combining theory and experiments to better understand the role of the stable actin pool in LTP. First, we test whether an experimentally constrained model^23^ without a stable pool exhibits altered actin dynamics or spine geometry at the timescale of the synaptic tag, and find that this is not the case. We then experimentally evaluate the fraction of stable actin 30, 90, and 150 minutes after chemically-induced LTP (cLTP), and find that it is increased by a factor of 2-3. Therefore, we adapt the theoretical model to include a dynamical stable pool and demonstrate that this refined model indeed exhibits altered spine geometry and actin dynamics on the timescale of hours, supporting the hypothesis that actin and spine geometry serve as a biophysical implementation of the synaptic tag.

## Results

### Model of dynamic actin and its modulation during LTP

We first test whether a model without a stable pool exhibits long-lasting perturbations of spine volume or actin dynamics after LTP. To this end, we exposed an existing model^22^, which is known to match the dynamic of actual spines^23^, to the characteristic changes in the concentrations of ABPs observed during LTP^15^.

**Fig. 1:**
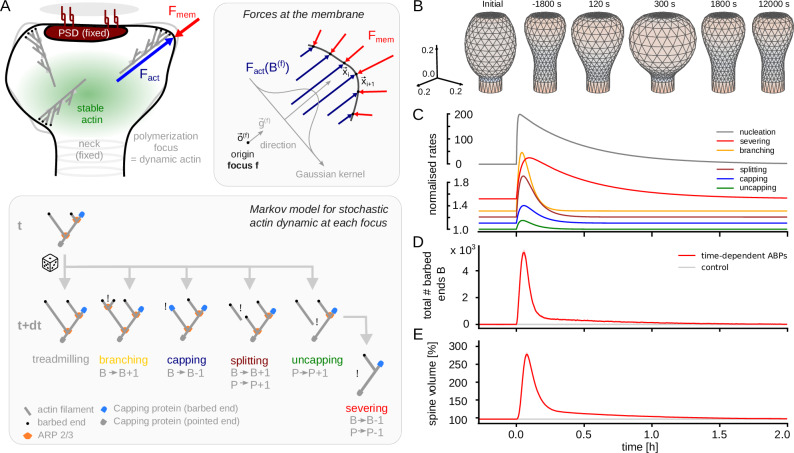
LTP-induced changes in actin dynamics and spine volume decay quickly. **A** Schematic of model components and relevant variables. **B** Example spine shapes from simulation. Colors indicate the curvature: blues for concave and reds for convex regions. **C** Normalized time-dependent rates of actin dynamics (upper scale bar applies for nucleation only). **D** Total number of barbed ends depicted as mean (solid curves) and standard deviation (shaded area) over 20 instances of the model. Red curves correspond to the model with LTP-induced changes from (**A**) and grey curves to a model without (**E**) Same for spine volume.

In the model, the spine membrane is described by a triangular mesh. Each point of the mesh, aside from points belonging to the PSD and the spine neck (Fig. 1A, top left), moves according to the local balance between forces exerted by the membrane and by the actin network (Fig. 1A, right panel). The membrane force is derived from the Canham-Helfrich free energy formalism and accounts for the membrane resisting against changes in spine volume, surface area and surface curvature (see Methods: Membrane force).

To model force from actin filaments, we assume that a spine contains a small number of actin polymerization foci^27^, each of them being a branched network of actin-filaments with a certain number of barbed ends *B*. The polymerization at these barbed ends leads to a retrograde flow of the connected filaments through the spine, which, in turn, generates a counter-force – for example by friction or breaking transiently formed bonds. Thus, each focus generates an expansive (outward directed) force, proportional to its number of barbed ends (Fig. 1A).

The number of barbed ends in each focus is modeled by a random process (Markov chain) in which the current state of a polymerization focus is described by the number of barbed ends *B* and the number of uncapped (exposed) pointed ends *P* (Fig. 1A, bottom panel). The actions of different ABPs are then assumed to occur at certain rates, and change the state of the focus accordingly. In particular we consider branching (*B* → *B* + 1) related to the ARP2/3 complex, capping (*B* → *B* − 1) related to the capping protein, severing and depolymerization (*P* → *P* − 1, *B* → *B* − 1) as well as splitting (*P* → *P* + 1, *B* → *B* + 1) both related to the activity of cofilin (see refs. ^10,28^ for a review). The number of barbed ends heavily fluctuates and ultimately reaches zero, leading to the removal of the respective focus. Accordingly, also new foci are nucleated randomly such that both the number of active foci and the number of barbed ends at each focus are stochastic. This enables the model to account for spine motility^23^. However, for quantitative analyses of our model, we run multiple simulations and provide average and standard deviations.

To model LTP, we use the relative concentration changes of the respective proteins in experiments^15^ where possible and multiply them with the basal rates in our model (Fig. 1C). Furthermore, new foci are nucleated randomly with a rate that is also transiently increased during LTP, due to the enhanced availability of actin monomers and increased cofilin concentrations^16^.

### Fast decay of LTP-induced perturbations in actin and spine geometry

We then simulated this model and tracked the time evolution of spine volume, number of foci and the sum of the number of barbed ends from all foci. In the beginning, the spine evolves from its initial condition (Fig. 1B, left) into its stationary state given by the membrane force (Fig. 1B, *t* = − 1800s). Upon the onset of the stimulation, we observe a rapid increase in the total number of actively polymerizing barbed ends in the spine (Fig. 1D) that determine the actin force in the model. However, this increased number of barbed ends decays at the same timescale as the altered rates of the ABPs (dominantly the branching and nucleation rate) and has mostly decayed after around 20-30 minutes. The volume of the spine increases by 150% within minutes (Fig. 1E), similar as observed in experiments^15,19^. Yet, after the initial increase, the volume undergoes approximately the same decay as the number of barbed ends (Fig. 1E), although somewhat more spread in time, as the spine membrane deforms more slowly. This is in line with previous findings that the spine size can be approximated a low-pass filtered version of the number of polymerization foci^22^, and, thus, also of the total number of barbed ends. However, as the model can reproduce large volume fluctuation on a timescale of seconds^23^, it is not surprising that it cannot preserve information about plasticity events on a longer timescale than a few minutes.

This is likely because our model only accounts for a dynamic pool of actin, which is located towards the spine tip and treadmilling very fast and not for the slower treadmilling actin pool—most likely cross-linked actin filaments—that experiments^14^ had shown. While these experiments already showed that cross-linked filaments are involved in the maintenance of LTP^14^, they did not quantify whether and how much the stable pool changes after LTP.

### Is there an increase in the stable pool after LTP?

To determine whether the LTP influences the size of the stable pool of actin on a timescale of hours, we proceeded to wet-lab experiments, focusing on hippocampal cultured neurons, a common model for neuroscience investigations. We used mature neurons (14 days in vitro) that expressed a fluorescently-tagged actin variant, carrying a GFP molecule, whose participation in functional reactions has been validated in the past (for example^29^, and references therein). We investigated the mobility of the actin molecules using fluorescence recovery after photobleaching (FRAP), a method in which the fluorescent proteins are bleached using a laser beam, in a specific area, and the entry of non-bleached molecules, from neighboring sides, is monitored. Such molecules replace the bleached ones, if they leave the respective area, and the fluorescence signal recovers, with a speed proportional to the molecule mobility^30^. However, if a stable population exists in the respective location, it will not be replaced by mobile molecules, since it does not leave the respective site (in spite of losing fluorescence by bleaching). Such a population is typically termed an immobile fraction, and would correspond, in our conditions, to a stably polymerized actin pool^14^.

**Fig. 2:**
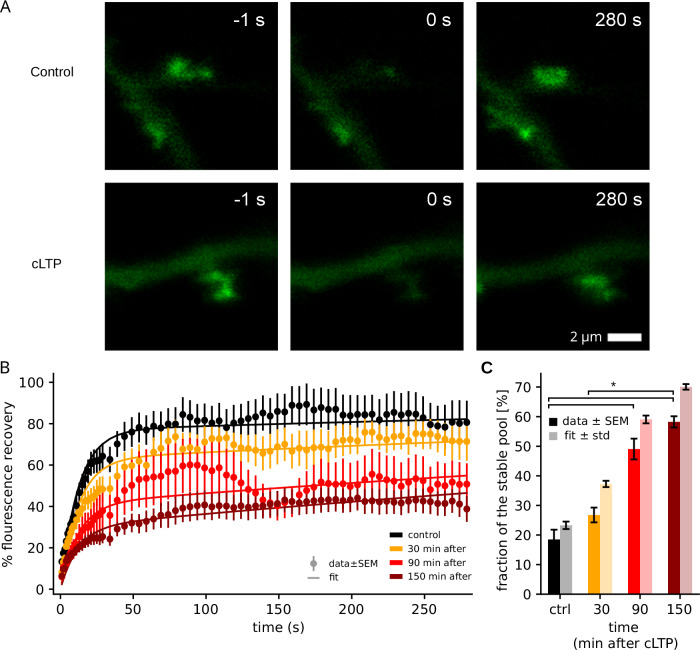
FRAP analysis of actin mobility in dendritic spines. **A** Examples of FRAP experiments performed either without (top panels, control) or 30 minutes after cLTP induction (bottom panels, cLTP). The images show representative dendritic spines before fluorescence photobleaching (left panels,  − 1 seconds), immediately afterwards (middle panels, 0 seconds) or after a recovery period (right panels, 280 seconds). **B** An analysis of the fluorescence recovery kinetics, indicated as means ± SEM (*N* = 16, 12, 9 and 11 spines for control and the cLTP experiments at increasing intervals as indicated by color; source data under^51^). Solid lines depict fitted double-exponential time-courses used for determining the stable fraction. **C** Bar graph of the the stable pool fractions in control as well as 30, 90 and 150 minutes after cLTP as measured by mean ± SEM of the immobile fractions obtained from the last 36 time points of the FRAP measurements in (**B**) (solid) and from a curve fit of the curves in (**B**) (transparent). The difference in the immobile fractions is significant (Kruskal-Wallis-test, *p* = 0.0056 with posthoc-Dunn-test *p* = 0.047, 0.008 and 0.047 for the three indicated significant differences).

We applied the FRAP procedure to postsynaptic spines, and determined the recovery of the fluorescence signal over approximately five minutes of imaging (Fig. 2A). We then performed the same experiment in cultures subjected to a chemical LTP procedure^31^. We observed a substantially higher immobile fraction at 30 minutes after LTP induction (approx. 35% vs. 25% in the control cultures, Fig. 2B) and find that this difference becomes even larger at 90 and 150 minutes after LTP (60–70%).

We conclude that there is a significant increase in the size of the stable pool within the first hours after LTP induction. Hence, modeling should account for the stable pool and its dynamic, to account for the stabilization of LTP.

### Modeling a stable actin pool

Our next step was therefore to add a time-variant stable pool to our model. This stable pool is assumed to be built-up by cross-linking with filaments from the dynamic pool at rate *k*_*bind*_ and decays by unbinding filaments with a rate *k*_*unbind*_. As a measure for the size of the dynamic pool, we use the total number of active barbed ends *B*_*tot*_, such that the dynamic of the stable pool *S* is given by

$$\frac{dS}{dt}={k}_{bind}(t){B}_{tot}-{k}_{unbind}(t)S$$

Note that the binding and unbinding rates are time-dependent as cross-linkers detach during the early phase of LTP and reattach after one to five minutes^15,32^. For simplicity, we model this by a step-function. Outside of this time window, the stable pool implements a low-pass filtered version of the dynamic actin. As some experiments do not report a decline in stable actin^14^, we also checked that the simulation results do not qualitatively differ if this unbinding period is left out (see Suppl. Fig. S5).

The stable pool also influences the actin force, as a larger stable pool in the spine leads to more resistance against the retrograde movement of the dynamic actin filaments. We implement this by multiplying the dynamic-pool-dependent actin force with ${\alpha }_{0}\left(1+q\,{f}_{S}\right)$, where *f*_*S*_ = *S*/(*S* + *B*_*tot*_) is the fraction of stable actin, *α*_0_ adjusts the overall scaling of the force, *q* scales the stable pool dependent contribution. In this way, the dynamic polymerization foci exert more force, when more stable actin is present, such that their joint action can enlarge the spine.

### Determining model parameters from experiment

**Fig. 3:**
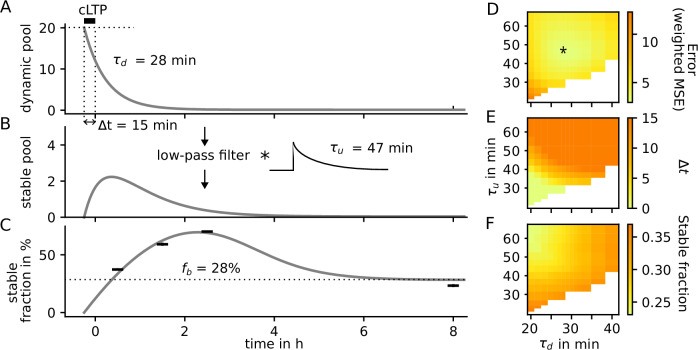
Adapting a coarse grained model to experiments. **A** Dynamic pool is assumed to decay exponentially after a fast, initial increase during LTP. This increase takes place at time Δ*t* before the end of the cLTP protocol. **B** Stable pool is assumed to be a low-pass filtered version of the dynamic pool. **C** The resulting fraction of stable F-actin exhibits an overshoot later than the stable pool itself. **D** Weighted mean squared error (MSE) for combinations of *τ*_*d*_ and *τ*_*u*_. Parameters of the best-fitting model (star) are used in (**A**-**C**). **E** Best matching time shift *Δt* and **F** best matching basal stable pool fraction *f*_*b*_ that were used to calculate errors in (**D**).

To determine the parameters of our newly introduced stable pool model, we investigate a course-grained, analytically treatable description of the two pools after LTP. In particular, we assume that—after the onset of the cross-linker binding—the stable actin pool starts at size zero and the dynamic pool exponentially decays towards its basal value (Fig. 3A). Furthermore, the cross-linker binding onset is assumed to occur at a time Δ*t* before the end of the cLTP protocol. Under these assumptions, we can analytically derive the time-course of the fraction of stable actin (Fig. 3B) as well as its fraction of the total F-actin (Fig. 3C). The latter can then be compared to immobile fraction in our FRAP-measurements to find the best matching model parameters. To this end, we tested different timescales for dynamic pool decay and cross-linker unbinding rate (axes in Fig. 3D–F), and optimize all other parameters (initial dynamic actin amplitude, time shift *Δt* and the basal stable pool fraction) within defined bounds to minimize the mean-squared error weighted by the variances of the measured values and (Fig. 3D). The optimal values of the time shift and the initial dynamic pool tend to lie at their upper bound (Fig. 3E), while the basal stable actin fraction takes intermediate values (Fig. 3F). The best fit between the time course of the stable actin fraction and the experimental data (Fig. 3C, star in Fig. 3D) is obtained when dynamic and static pool decay at 28 minutes and 47 minutes, respectively (star in Fig. 3D). Accordingly we use these timescales for the decay of the nucleation rate and the unbinding rate of cross-linkers and examine the behavior of the full spine geometry model.

### Reproduction of overshoot in stable actin ratio

**Fig. 4:**
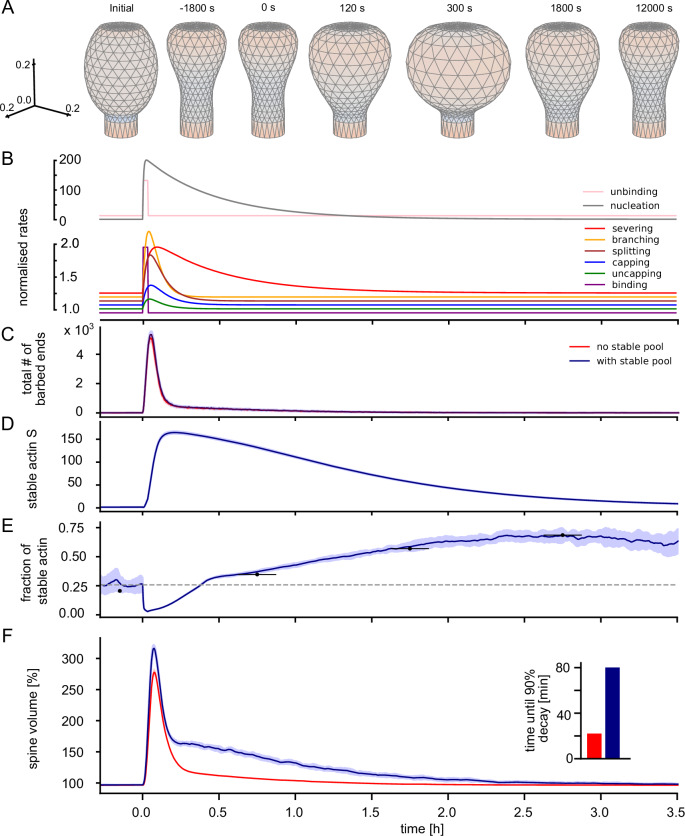
LTP-induced changes in actin dynamics and spine volume persist longer with stable pool. **A** Example spine shapes at different time-points as indicated on top. At *t* = 1800s an increased size is still visible, whereas it has decayed at 12000s. **B** Time-course of normalized rates determining actin dynamics (upper scale bar applies for unbinding and nucleation). **C** Total number of barbed ends in the spine (summed over all polymerization foci). Blue curves show simulations using the the novel stable pool, and red curves the model without the stable pool equivalent to *q* = 0, compare Fig. 1). Curves depict mean (solid) and standard deviation (shaded) over 20 simulations of the stochastic actin dynamics. **D** Time-course of the stable pool. **E** Time-course of the fraction of actin allocated to stable pool. **F** Time-course of the spine volume. With the stable pool, a slowly decaying component of the spine volume is clearly observable. (Inset) Time until 90% of the volume increase have decayed for models with and without stable pool. The time-course of further model quantities is depicted in Supp. Figs. 1 and 6.

With the stable pool integrated into the model, we simulated the actin dynamic and temporal evolution of spine geometry after LTP. Again, we observe that the time dependent reaction rates after a stimulus (Fig. 4B) induce a large growth in the total number of barbed ends (Fig. 4C). The stable pool drops to very low values once the cross-linkers unbind, but recovers quickly once the reattachment commences (Fig. 4D).

Strikingly, at that time-point, the amount of dynamic actin filaments is much larger than the sum of dynamic and stable actin under basal conditions. The stable pool growth is proportional to the amount of dynamic actin and aims to reestablish the basal ratio between the pools. Thus, the stable pool grows beyond its basal level and exhibits a significant overshoot. After the dynamic pool decays, this overshoot becomes also visible in the stable pool fraction.

In the following, this overshoot decays at the slow timescale of the stable pool such that it is visible for 1 to 2 hours. We also evaluated the experimentally measured fraction between stable and total filamentous actin (i.e. stable and dynamic). To calculate the dynamic actin, we use a moving average with a 300s window on the number of barbed ends, which prevents the fraction to undergo large fluctuations. We find that also the fraction of stable actin exhibits an overshoot, although much later than the stable pool itself (Fig. 4E). This is because the dynamic pool starts higher but decreases faster than the stable pool. The resulting overshoot of the stable pool fraction matches the experimental measurements (Fig. 4E), when using the time-shift fitted above.

The consequence of the overshoot in stable pool is a longer-lasting significant increase in spine volume (Fig. 4F, grey markers). Hereby, the decay of the LTP-induced increase in spine volume does not seem to follow an exponential decay with a single timescale, but rather has a slow and a fast component. When evaluating the time until 90% of the LTP-induced increase has decayed, we consequently observe a much longer timescale of around 80 minutes as compared to around 20 minutes for the model without stable pool (Fig. 4F, inset). We therefore conclude, that the stable actin pool could provide a transient memory of a plasticity event that effects spine geometry for more than two hours—that is, on the timescale of the synaptic tag.

## Discussion

Using a combined approach of experiments, simulations and theory, we have demonstrated that the stable pool of actin is enlarged for at least 2.5 hours after an LTP-like stimulus, which is in line with a long-lasting up-regulation of the F-actin content of the spine observed in FRET-experiments^33^.

Parts of this excess stable actin could correspond to the so called the enlargement pool—a filament pool with intermediate stability and decreased retrograde movement—which must be retained within the spine for lasting LTP^14^. This pool could emerge from filaments which are not yet fully cross-linked or missing additional molecules to become stable (e.g., cofilin^13^, cortactin^11^). Yet, with a typical lifetime of around 8 minutes^14^, the dynamic of this pool should not be relevant at the time points considered in our experiments (given it remains contained in the spine). In our model, we are only using a single differential equation for all cross-linked or otherwise stabilized actin filaments and, thus, cannot account for the faster decay of the enlargement pool or its containment. Instead, we assume that it is converted into stable actin on the timescales relevant for this study. In general, the formation of the stable pool is likely a graded, spatially inhomogeneous process involving crosslinkers and other ABPs and leading to different stability of the filaments at different locations, which is only very coarsely approximated by two (or more) pools. In particular, multiple crosslinking proteins such as drebrin, cortactin^11^, CaMKII^12,16^ or myosins could be active at the same time and also the action of ABPs like cofilin^13^ could all contibute to actin stabilization. To model this in more detail, continuous fields for the degree of cross-linking and the stability of the filaments could be introduced, which would have to be calibrated by longitudinal superresolution data on the distribution of all involved proteins. As the current study focuses on temporal dynamics on the timescale of hours, we abstract this spatial (and temporal) inhomogeneity using a single dynamic equation.

Given this dynamic, we argue that the stable actin pool could serve as a biophysical implementation of the synaptic tag, as it fulfills all respective criteria^5,7^: First, a tag needs to be specific for the potentiated synapse, which certainly is the case for the stable pool, as the cross-linked actin filaments stay localized within the spine. This is also supported by our FRAP experiments (Fig. 2). Secondly, the tag should not require protein synthesis. This is also the case for the stable pool, which relies on an increased polymerization of the existing actin molecules. Along this line, actin is one of the most stable proteins in the synapse (half-live of 17-19 days in the mouse brain, more than twice longer than the median value for all proteins^34^;) and there is, to our knowledge, no evidence that actin is synthesized as a plasticity-related protein. Again, this is supported by our FRAP experiments in which we block protein synthesis through anisomycin. Third the tag should decay on a timescale of a few hours, which is the case for the increased volume and the overshoot of the stable pool. This finding, of course, heavily relies on the timescales used in the model, which we obtained from fitting the FRAP results. Here, the increased activity of the dynamic pool for around 30 minutes corresponds to the increased concentrations of actin binding proteins such as cofilin^15^. The timescale of the stable pool decay (47 minutes) is on the same order of magnitude as the molecular exchange in stable actin filaments (17 minutes reported in ref. ^14^), but a little longer as it does not model molecular exchange but the net-loss of filament through treadmilling (which also includes growth). Fourth, the tag should be able to recruit newly-synthesized proteins. As stable actin affects the geometry of the spine, it will also regulate the space available to reorganize the PSD. An expansion of the spine transiently decreases the concentration of PSD proteins^15^, which, in turn, alters their clustering behavior as well as the PSD structure via liquid-liquid phase separation on a longer timescale^35^. Moreover, the PSD itself interacts with actin^36,37^ and consequently also with the stable pool. More stable actin may thus lead to the recruitment of more PSD proteins. Thus, while our model does not yet include such a protein capture mechanism, it is reasonable to assume that protein capture will indeed occur^38^. However, these hypotheses have to be tested rigorously in future work.

Apart from its role for a synaptic tag, the inclusion of a dynamic stable actin pool is a novelty in comparison to previous models^20,22–24^, which have largely neglected the time-evolution of the stable pool. Yet, as also these models rely on barbed end numbers or densities, the modifications presented here for the stable pool could be similarly applied to them. Functionally, our stable pool acts as a slow (low-pass filtered) version of the fast dynamic actin. Synapses with such fast-and-slow dynamics have been shown to exhibit many properties of brain-like learning and memory^39^ and may help with continual learning^40^. Accordingly, the synapse model we introduced can be assumed to exhibit similar beneficial characteristics for learning and memory.

This study focuses on dynamics within a single spine. However, spines continuously interact with other synaptic constituents and their extracellular environment, which may influence the spine-internal dynamics. Our model provides a good basis to investigate this: On the one hand, the interaction with the pre-synapse may give rise to interesting dynamics. The PSD and the presynapse are linked by diverse trans-membrane proteins (such as neurexins and neuroligins), which can mediate signaling between the two. Thereby, presynaptic structures (for example the vesicle cluster^41^) and postsynaptic structures can mechanically interact with each other. This can, in turn, regulate synaptic function. For example, morphological changes induced by mechanically pushing the presynapse increase vesicle release^42^. Thus, the deformation of the post-synapses studied here may also contribute to the fast up-regulation of synaptic weights during early LTP and possibly also short-term plasticity^43^. To investigate this, a suitable model of the mechanics of the presynapse will have to be devised by future research. On the other hand, spine growth during LTP can push away glia or the extracellular matrix. This, in turn may leave “holes" in the surrounding into which spines can more easily extend and into which spines could regrow more easily^44^. Also, spine enlargement might be aided and perpetuated by anchoring the spine membrane to the surrounding structures by cell adhesion molecules such as integrins or cadherins^45–47^, which could add another level of dynamic complexity^48^. In our model, such interaction could be included by adjusting the deformation speed according to the location and deformation history, or even adding further force sources and it can be expected that such interactions may give rise to another slow dynamics that may aid long-term memory.

Yet, as these surrounding elements and their force contributions are currently not included, the forces from actin in our model needs to be smaller than in reality to obtain realistic spine deformations. In particular, in our model, the force amplitude for the actin filaments is *α*_0_ = 0.5*f* *N* and the summed force towards the presynapse reach around 0.1*nN* (see Supp. Fig. 6). Previous works have estimated the force from single filaments to lie in the range of a few pN^49^ which can, during LTP, result in summed forces up to 10nN. We assume that, when including the spines surrounding, such higher force values will also be required in our model.

At the current stage, however, we can already draw conclusions about postsynaptic processes. Here, our work strongly suggests that interaction between spine geometry and time-dependent actin polymerization and cross-linking plays an essential role in the consolidation of synaptic plasticity and memory, by providing a long-lasting, but transient memory of past stimuli, which is consistent with a biophysical implementation of the synaptic tag.

## Methods

### Experimental model and study participant details

We used newborn rats (*Rattus norvegicus*) to prepare cultures combining cells from the entire litter (male and female). We have complied with all relevant ethical regulations for animal use: Animals were handled according to the regulations of the local authorities, the University of Göttingen and the State of Lower Saxony (Landesamt für Verbraucherschutz, LAVES, Braunschweig, Germany). All animal experiments were approved by the local authority, the Lower Saxony State Office for Consumer Protection and Food Safety (Niedersächsisches Landesamt für Verbraucherschutz und Lebensmittelsicherheit) and were performed in accordance with the European Communities Council Directive (2010/63/EU).

### Experimental procedures: wet lab

#### Rat hippocampal cultures

We prepared dissociated cultures from hippocampi following procedures established before, exactly as described in ref. ^29^. In brief, hippocampi (from newborn wild-type, Wistar animals) were dissected in HBSS (Hank’s Buffered Salt Solution, 140 mM NaCl, 5 mM KCl, 4 mM, 6 mM glucose, NaHCO_3_, 0.4 mM KH_2_PO_4_ and 0.3 mM Na_2_HPO_4_). They were then incubated with an enzyme solution, prepared in DMEM (Dulbecco’s Modified Eagle Medium, #D5671, Sigma-Aldrich, Germany), for 60 minutes. The enzyme cocktail contained 50 mM EDTA, 100 mM CaCl_2_,0.5 mg/mL cysteine and 2.5 U/mL papain, and was carbogen-saturated for 10 min before application. The materials were then exposed to an enzyme-deactivation buffer (DMEM with 0.2 mg/mL bovine serum albumin, 0.2 mg/mL trypsin inhibitor and 5 percent FCS, fetal calf serum). After cellular trituration, we seeded the material on 18mm circular coverslips, at 80,000 cells per coverslip. The coverslips were prepared by treating with nitric acid, followed by sterilization and exposure to 1 mg/mL poly-L-lysine, overnight. The cells adhered to the coverslips for 1 − 4h at 37 °C DMEM containing horse serum (10 percent), 2 mM glutamine and 3.3 mM glucose. This medium was then replaced with Neurobasal-A (Life Technologies, Carlsbad, CA, USA) containing 2% B27 (Gibco, Thermo Fisher Scientific, USA) supplement, 1% GlutaMax (Gibco, Thermo Fisher Scientific, USA) and 0.2% penicillin/streptomycin mixture (Biozym Scientific, Germany). The coverslips were incubated at 37 °C, under 5% CO_2_ atmosphere, until use.

DIV 4 primary hippocampal neurons were transfected in order to express GFP-tagged actin, using Lipofectamine™ 2000 Transfection Reagent (Invitrogen™) as vehicle.

#### Chemical LTP induction

The procedure of inducing chemical LTP was adapted from the methods described by Zheng and collaborators^31^. Briefly, DIV15 neurons were washed once with a basal buffer (150 mM NaCl, 5 mM KCl, 2 mM CaCl_2_, 2 mM MgCl_2_, 10 mM HEPES, 30 mM D-Glucose, pH=7.34-7.36) to get rid of the debris from the cell media. Then, neurons were washed twice with Mg^2+^-free buffer (150 mM NaCl, 5 mM KCl, 2 mM CaCl_2_, 10 mM HEPES, 30 mM D-Glucose, 0.02mM bicuculline and 0.001 mM picrotoxin, pH=7.34-7.36), for a total of 5 minutes. Neurons were further incubated at 37 °C in a glycine-supplemented buffer (150 mM NaCl, 5 mM KCl, 2 mM CaCl_2_, 10 mM HEPES, 30 mM D-Glucose, 0.02 mM bicuculline, 0.001 mM picrotoxin and 0.2 mM glycine, pH=7.34-7.36) for 15 minutes. Following a wash with the basal buffer, the neurons were incubated at 37 °C in Mg^2+^ buffer (150 mM NaCl, 5 mM KCl, 2 mM CaCl_2_, 2 mM MgCl_2_, 10 mM HEPES, 30 mM D-Glucose, 0.02 mM bicuculline and 0.001 mM picrotoxin, pH=7.34-7.36) for 30 minutes, prior to live imaging in basal buffer.

#### Neuronal live imaging

In order to track spine enlargement induced by cLTP, live neurons were imaged every 5 minutes (during cLTP) and every 15 minutes for 2 hours (after cLTP). A TCS SP5 confocal microscope (Leica, Wetzlar, Germany) equipped with an HCX Plan Apochromat 100 × 1.40 oil immersion objective was used for the imaging. The 488 nm wavelength of an Argon laser was used for imaging of GFP.

Since we aim to assess the decay of the tag independently of protein synthesis, we decided to block protein synthesis 10 minutes before starting cLTP by the usage of the specific inhibitor anisomycin (0.13 μM, A9789, Sigma-Aldrich, Germany). Anisomycin was applied in both cLTP and control conditions.

Furthermore, in order to investigate the persistence of the stable F-actin pool, FRAP recordings were generated immediately after the incubation after cLTP (here reported as 30min after induction) as well as either 1 hour or 2 hours after (reported as 90 and 150 minutes after cLTP). Individual neuronal spines were used for the FRAP (Fluorescence Recovery After Photobleaching) experiments. Before bleaching, four images were taken, and then, the region of interest was bleached for 100 ms. The bleaching intensity was defined as in ref. ^29^: 50 μW, at 488 nm. After bleaching, 10 images were taken every 1 s, then 10 images every 2 s, and 50 images every 5 s. For the control condition, DIV15 hippocampal neurons were shortly washed with the basal buffer and directly live-imaged, as described above.

In both mentioned experimental designs, all the steps required for cLTP induction were performed on the microscope stage.

#### FRAP image analysis

The FRAP movies were analyzed using a custom-written routine in MATLAB (The MathWorks Inc, Natick, MA, USA). The images were loaded, and were then automatically aligned, and the FRAP area was determined automatically, by comparing the last frame before bleaching to the first one post-bleaching, and was set as the FRAP region of interest (ROI). The ROI intensity was then determined for all of the frames, was corrected for background, by subtracting the average intensity signals in other, non-cellular areas, and was then normalized to the pre-bleaching intensity. The resulting FRAP curves were then averaged and displayed as mean and SEM in Fig. 2.

FRAP experiments have been conducted for *N* = 16, *N* = 12, *N* = 9 and *N* = 11 (each from an independent FRAP experiment) for the control and cLTP experiments with increasing time difference, respectively. As described, values were normalized by the basal fluorescence of each spine and then depicted by their mean and standard error of the mean.

The experimentally-derived immobile (stable) fractions represent the % of the initial intensity of the spines that did not recover by the end of the recording. This definition fits well with the observation that the recovery curves saturated within our observation time.

The fractions of the stable pool were also analyzed by fitting an exponential decay

$$F(t)=f(1-\exp (-t/{\tau }_{{{{\rm{stable}}}}}))+(1-f)\cdot (1-\exp (-t/{\tau }_{{{{\rm{dynamic}}}}}))$$

to all four curves simultaneously using pythons curve_fit function. Here, $f={(\,{f}_{b},{f}_{30},{f}_{90},{f}_{150})}^{T}$ are the fractions of stable actin and *τ*_dynamic_ and *τ*_stable_ the timescale of the dynamic and stable pool, respectively. The standard deviation of the fractions was obtained from the covariance matrix of the fit parameters.

### Biophyiscal model of membrane dynamics

#### Membrane model and initialization

The spine membrane is modeled with a 3D triangular mesh. It is initialized as a sphere with a radius of 0.25 μm and 20 segments along the latitudinal and longitudinal direction. Every ring along the latitudinal axis is alternatingly rotated by  ± 9°, to obtain regular triangles. We then flatten the top four rings of vertices to the level of the fourth ring and declare these points as the PSD. Further, to model the spine neck, we replace the bottom 2 rings by a cylinder with the same radius as the second-last ring, and a length of 0.1. Afterwards, the *x* and *y* coordinates of all points are scaled by a factor of 0.7 to obtain an initial configuration corresponding to a thin mushroom-shaped spine.

#### Membrane dynamic

The vertices of the PSD and the neck remain fixed, as these regions are supported by either transmembrane proteins like neurexins/neuroligins (PSD) or by a rigid ring-structure of actin filaments (neck). At all other locations, a vertex *i*’s position ${\overrightarrow{x}}_{i}$ changes according to the forces generated by the actin filaments ${\overrightarrow{F}}_{act,i}$ and the forces generated by the membrane ${\overrightarrow{F}}_{mem,i}$ at that vertex. For numerical stability, we added a force ${\overrightarrow{F}}_{tan,i}$, which moves vertices along the spine surface to preserve shape but obtain an approximately equilateral mesh. The movement of vertex *i* is then given by:

$$\frac{d{\overrightarrow{x}}_{i}}{dt}=\zeta ({\overrightarrow{F}}_{mem,i}+{\overrightarrow{F}}_{act,i}+{F}_{tan,i}).$$

The following sections detail the calculations of all three forces.

#### Membrane force

Following previous works (^22–24^, see ref. ^50^ for a review), we derive the membrane force from the Canham-Helfrich free energy

$$E=pV+\sigma A+\frac{\kappa }{2}\int\,{H}^{2}dA$$

where *p* is the pressure difference between inside and outside the membrane, V is the spine (head) volume, *σ* the elasticity of the membrane, *A* its area, *κ* the membrane bending modulus and *H* the mean curvature. Note that, for simplicity, we assume constant pressure and membrane elasticity and do not model how these parameters may change through the deformation of the spine, membrane flow from the dendritic shaft, exocytosis of vesicles, or other cellular membrane sources.

The force at vertex *i* is the derivative of this energy with respect to the (three dimensional) position of the mesh vertices ${\overrightarrow{x}}_{i}$:

$${\overrightarrow{F}}_{mem,i}=\frac{\partial E}{\partial {\overrightarrow{x}}_{i}}=p\frac{\partial V}{\partial {\overrightarrow{x}}_{i}}+\sigma \frac{\partial A}{\partial {\overrightarrow{x}}_{i}}+\frac{\partial }{\partial {\overrightarrow{x}}_{i}}\frac{\kappa }{2}\int\,{H}^{2}dA$$

where $\partial /\partial {\overrightarrow{x}}_{i}={\overrightarrow{\nabla }}_{{\overrightarrow{x}}_{i}}={(\partial /\partial {x}_{i,1},\partial /\partial {x}_{i,2},\partial /\partial {x}_{i,3})}^{T}$ is the derivative with respect to the individual coordinates of vertex *i*

In the following we will derive approximations for the three terms on the right hand side for our mesh:

- **Volume:** The volume can be calculated as
  $$V=\frac{1}{6}{\sum}_{m=\{i,j,k\}}({\overrightarrow{x}}_{i}\times {\overrightarrow{x}}_{j})\cdot \overrightarrow{{x}_{k}}$$
  where the sum runs over all triangles *m* with points *i*, *j* and *k*. For the contribution of the individual triangle *V*_*m*_, the derivatives are
  $$\frac{\partial {V}_{m}}{\partial \overrightarrow{{x}_{i}}}=\frac{1}{6}({\overrightarrow{x}}_{j}\times {\overrightarrow{x}}_{k})\quad \frac{\partial {V}_{m}}{\partial \overrightarrow{{x}_{j}}}=\frac{1}{6}({\overrightarrow{x}}_{k}\times {\overrightarrow{x}}_{i})\quad \frac{\partial {V}_{m}}{\partial \overrightarrow{{x}_{k}}}=\frac{1}{6}({\overrightarrow{x}}_{i}\times {\overrightarrow{x}}_{j})$$
  In the source code, we calculate these three contributions for each triangle and then add them to the summed force of the respective vertices *i*, *j* and *k*.
- **Area:** The area can be calculated as
  $$A=\frac{1}{2}{\sum}_{m=\{i,j,k\}}| ({\overrightarrow{x}}_{k}-{\overrightarrow{x}}_{i})\times ({\overrightarrow{x}}_{j}-{\overrightarrow{x}}_{i})| .$$
  Consider triangle sides $\overrightarrow{a}={\overrightarrow{x}}_{k}-{\overrightarrow{x}}_{i}$ and $\overrightarrow{b}={\overrightarrow{x}}_{j}-{\overrightarrow{x}}_{i}$, then
  $${A}_{m}=1/2| \overrightarrow{b}\times \overrightarrow{a}| 	 = 1/2\sqrt{(\overrightarrow{b}\times \overrightarrow{a})(\overrightarrow{b}\times \overrightarrow{a})} \\ 	=1/2\sqrt{(\overrightarrow{b}\cdot \overrightarrow{b})(\overrightarrow{a}\cdot \overrightarrow{a})-{(\overrightarrow{a}\cdot \overrightarrow{b})}^{2}}$$
  through the Binet-Cauchy-identity. We know that $\frac{\partial \overrightarrow{a}}{\partial \overrightarrow{{x}_{k}}}=1$ and $\frac{\partial \overrightarrow{b}}{\partial \overrightarrow{{x}_{k}}}=0$, such that we can calculate the derivative as
  $$\frac{\partial {A}_{m}}{\partial {\overrightarrow{x}}_{k}}	= \frac{\partial {A}_{m}}{\partial \overrightarrow{a}}=1/4\frac{1}{{A}_{m}}\left(2(\overrightarrow{b}\cdot \overrightarrow{b})\overrightarrow{a}+2\left(\overrightarrow{a}\cdot \overrightarrow{b}\right)\overrightarrow{b}\right) \\ 	 =\frac{1}{2{A}_{m}}\left(| \overrightarrow{b}{| }^{2}\overrightarrow{a}+\left(\overrightarrow{a}\cdot \overrightarrow{b}\right)\overrightarrow{b}\right)$$
  In our implementation, we calculate the area contributions by iterating through all triangles. Due to symmetry reasons, we do not calculate the derivatives w.r.t ${\overrightarrow{x}}_{i}$ and ${\overrightarrow{x}}_{j}$, but just calculate the above contribution for each cyclic permutation of *i*, *j* and *k* and add this contribution to the area-dependent force of the respective vertex.
- **Curvature:** We approximate the curvature integral (different from^22^, but see^23^) as
  $$\frac{\kappa }{2}\int\,{H}^{2}dA\approx \sqrt{3}\kappa {\sum}_{(m,n)}\left(1-\underbrace{{\overrightarrow{n}}_{m}\cdot {\overrightarrow{n}}_{n}}_{\cos {\theta }_{mn}}\right)$$
  with *m* and *n* being adjacent surfaces of the mesh and ${\overrightarrow{n}}_{m}$ and ${\overrightarrow{n}}_{n}$ their (unit length) face normals. These normals, in turn, depend on the position of the vertices spanning the triangle.
  In the following, we assume that the points *i*, *j* and *k* span a triangle *m* and *l* is the third point in the neighboring triangle *n* containing *i* and *j* such that we can use side $\overrightarrow{b}={\overrightarrow{x}}_{j}-{\overrightarrow{x}}_{i}$ twice. The (unit length) face normals are then
  $${\overrightarrow{n}}_{m}= 	 \frac{({\overrightarrow{x}}_{k}-{\overrightarrow{x}}_{i})\times ({\overrightarrow{x}}_{j}-{\overrightarrow{x}}_{i})}{| | ({\overrightarrow{x}}_{k}-{\overrightarrow{x}}_{i})\times ({\overrightarrow{x}}_{j}-{\overrightarrow{x}}_{i})| | }=:\frac{(\overrightarrow{a}\times \overrightarrow{b})}{| | \overrightarrow{a}\times \overrightarrow{b}| | } \,{{\mbox{and}}}\,{\overrightarrow{n}}_{n} \\ = 	\frac{({\overrightarrow{x}}_{j}-{\overrightarrow{x}}_{i})\times ({\overrightarrow{x}}_{l}-{\overrightarrow{x}}_{i})}{| | ({\overrightarrow{x}}_{j}-{\overrightarrow{x}}_{i})\times ({\overrightarrow{x}}_{l}-{\overrightarrow{x}}_{i})| | }=:\frac{\overrightarrow{b}\times \overrightarrow{d}}{| | \overrightarrow{b}\times \overrightarrow{d}| | }.$$
  We apply Binet-Cauchy to the numerator ($(\overrightarrow{a}\times \overrightarrow{b})\cdot (\overrightarrow{b}\times \overrightarrow{d})=(\overrightarrow{a}\cdot \overrightarrow{b})(\overrightarrow{b}\cdot \overrightarrow{d})-(\overrightarrow{a}\cdot \overrightarrow{d})| \overrightarrow{b}{| }^{2}$) and to the denominator ($| | \overrightarrow{a}\times \overrightarrow{b}| {| }^{2}=| \overrightarrow{a}{| }^{2}| \overrightarrow{b}{| }^{2}-{(\overrightarrow{a}\cdot \overrightarrow{b})}^{2}$) obtaining a product of denominators
  $$N(\overrightarrow{a},\overrightarrow{b},\overrightarrow{d}):=\sqrt{{(| \overrightarrow{a}{| }^{2}| \overrightarrow{b}{| }^{2}-{{(\overrightarrow{a}\cdot \overrightarrow{b})^{2}}})}{(| \overrightarrow{b}{| }^{2}| {\overrightarrow{{d}^{2}}}| -{(\overrightarrow{b}\cdot \overrightarrow{d})^{2}})}}.$$
  With this, we can easily evaluate the derivatives:
  $$\frac{\partial \cos {\theta }_{mn}}{\partial \overrightarrow{a}} = \, 	\frac{1}{N(\overrightarrow{a},\overrightarrow{b},\overrightarrow{d})}\left(\overrightarrow{b}(\overrightarrow{b}\cdot \overrightarrow{d})-\overrightarrow{d}| \overrightarrow{b}{| }^{2}\right) \\ 	 - \frac{(\overrightarrow{a}\cdot \overrightarrow{b})(\overrightarrow{b}\cdot \overrightarrow{d})-(\overrightarrow{a}\cdot \overrightarrow{d})| \overrightarrow{b}{| }^{2}}{N{(\overrightarrow{a},\overrightarrow{b},\overrightarrow{d})}^{3}} \\ 	 (\overrightarrow{a}| \overrightarrow{b}{| }^{2}-\overrightarrow{b}(\overrightarrow{a}\cdot \overrightarrow{b}))(| \overrightarrow{b}{| }^{2}| \overrightarrow{d}{| }^{2}-{(\overrightarrow{b}\cdot \overrightarrow{d})}^{2}) \\ \frac{\partial \cos {\theta }_{mn}}{\partial \overrightarrow{b}}= 	\frac{1}{N(\overrightarrow{a},\overrightarrow{b},\overrightarrow{d})}\left(\overrightarrow{a}(\overrightarrow{b}\cdot \overrightarrow{d})+\overrightarrow{d}(\overrightarrow{a}\cdot \overrightarrow{b}) -2(\overrightarrow{a}\cdot \overrightarrow{d})\overrightarrow{b}\right) \\ 	 -\frac{(\overrightarrow{a}\cdot \overrightarrow{b})(\overrightarrow{b}\cdot \overrightarrow{d})-(\overrightarrow{a}\cdot \overrightarrow{d})| \overrightarrow{b}{| }^{2}}{N{(\overrightarrow{a},\overrightarrow{b},\overrightarrow{d})}^{3}} \\ 	 \left[(\overrightarrow{b}| \overrightarrow{a}{| }^{2}-\overrightarrow{a}(\overrightarrow{a}\cdot \overrightarrow{b}))(| \overrightarrow{b}{| }^{2}| \overrightarrow{d}{| }^{2}-{(\overrightarrow{b}\cdot \overrightarrow{d})}^{2})\right. \\ 	 + \left.(| \overrightarrow{a}{| }^{2}| \overrightarrow{b}{| }^{2}-{(\overrightarrow{a}\cdot \overrightarrow{b})}^{2})\left.\right)(\overrightarrow{b}| \overrightarrow{d}{| }^{2}-\overrightarrow{d}(\overrightarrow{b}\cdot \overrightarrow{d}))\right] \\ \frac{\partial \cos {\theta }_{mn}}{\partial \overrightarrow{d}}=	\frac{1}{N(\overrightarrow{a},\overrightarrow{b},\overrightarrow{d})}\left(\overrightarrow{b}(\overrightarrow{a}\cdot \overrightarrow{b})-\overrightarrow{a}| \overrightarrow{b}{| }^{2}\right) \\ 	 -\frac{(\overrightarrow{a} \cdot \overrightarrow{b})(\overrightarrow{b}\cdot \overrightarrow{d})-(\overrightarrow{a}\cdot \overrightarrow{d})| \overrightarrow{b}{| }^{2}}{N{(\overrightarrow{a},\overrightarrow{b},\overrightarrow{d})}^{3}} \\ 	 (| \overrightarrow{a}{| }^{2}| \overrightarrow{b}{| }^{2}-{(\overrightarrow{a}\cdot \overrightarrow{b})}^{2})\left.\right)\left(\overrightarrow{d}| \overrightarrow{b}{| }^{2}-\overrightarrow{b}(\overrightarrow{b}\cdot \overrightarrow{d})\right.$$
  Then, with ${E}_{mn}=\tilde{\kappa }(1-cos{\theta }_{mn})$ and $\frac{\partial \overrightarrow{a}}{\partial {\overrightarrow{x}}_{i}}=-1,\frac{\partial \overrightarrow{a}}{\partial {\overrightarrow{x}}_{j}}=0$ etc., the derivatives w.r.t. each vertex are:
  $$\frac{\partial {E}_{mn}}{\partial {\overrightarrow{x}}_{i}}=\tilde{\kappa }\left(\frac{\partial \cos {\theta }_{mn}}{\partial \overrightarrow{a}}+\frac{\partial \cos {\theta }_{mn}}{\partial \overrightarrow{b}}+\frac{\partial cos{\theta }_{mn}}{\partial \overrightarrow{d}}\right),$$
  $$\frac{\partial {E}_{mn}}{\partial {\overrightarrow{x}}_{j}}= 	 \tilde{\kappa }\left(-\frac{\partial \cos {\theta }_{mn}}{\partial \overrightarrow{b}}\right), \frac{\partial {E}_{mn}}{\partial {\overrightarrow{x}}_{k}}=\tilde{\kappa }\left(-\frac{\partial \cos {\theta }_{mn}}{\partial \overrightarrow{a}}\right),$$
  and
  $$\frac{\partial {E}_{mn}}{\partial {\overrightarrow{x}}_{l}}=\tilde{\kappa }\left(-\frac{\partial \cos {\theta }_{mn}}{\partial \overrightarrow{d}}\right)$$
  In the code, the vectors $\overrightarrow{a}$, $\overrightarrow{b}$, $\overrightarrow{d}$ as well as the scalars $\overrightarrow{a}\cdot \overrightarrow{b}$, $\overrightarrow{b}\cdot \overrightarrow{d}$, $\overrightarrow{a}\cdot \overrightarrow{d}$ and $N(\overrightarrow{a},\overrightarrow{b},\overrightarrow{d})$ are pre-calculated for all triangles before the force contributions are calculated and assigned to the respective vertices.
  These terms signify the force contribution of triangles *m* and *n* (that share the edge *ij*) to the vertices *i*, *j*, *k*, *l*. By evaluating them for all edges and summing the force contributions from different triangle constellations for each vertex, we obtain the total curvature-induced force for each vertex.

#### Tangential force and mesh equilibration

The above described numerical approximations work best if all involved triangle edges are approximately equal. Therefore, we induce an additional tangential force. For this, we first sum the vectors of all edges emerging from a vertex *i* into a sum-vector *S*_*i*_. Thus, if the edges are not equal, the sum vector will point towards the long edges, where the point should be moved. We then determine to which of triangles containing point *i* the sum-vector can be projected (positive scalar products with both triangle sides starting at *i*) and project it to that plane. We finally multiply the sum vector by a factor of 10*pN*/*μm* to obtain a tangential force ${\overrightarrow{F}}_{tan,i}$. Note, this tangential force moves the vertices only along the existing surface but still can slightly change volume, area and curvature, especially for strongly curved local neighborhoods.

### Actin dynamics and forces

Actin forces are generated from distinct polymerization foci within the spine^22,27^. Hence our model allows for multiple foci of actin activity which act on the membrane at different points.

#### Dynamic of one focus

We abstract the complex growth of the filament tree to a Markov process with two state variables: the number of barbed ends *B* and the number of uncapped (exposed) pointed ends *P*. During the simulation, there are multiple transitions which can take place corresponding to the actions of various actin binding proteins:

- *Branching*(*B* → *B* + 1): Due to the attachment of ARP2/3 a new filament can branch off an existing one which gives rise to a new barbed end. The rate at which this happens is
  $${\gamma }_{branch}(t)=\phi {k}_{on}\delta a\exp \left(-\frac{| | {F}_{mem}(t)| | \delta }{{k}_{B}TB(t)}\right)\frac{1}{B(t)}$$
  where *ϕ* is a proportionality constant, *k*_*on*_ is the G-actin assembly rate constant, *a* is the available actin concentration, *F*_*mem*_ is the counteracting membrane force, *δ* is the length of G-actin, *k*_*B*_*T* is the thermal energy, and *B*(*t*) the number of barbed ends at time t.
- *Capping* (*B* → *B* − 1): When a capping protein (e.g. CapZ) attaches to a barbed end, it stops further polymerization, essentially removing it.
- *Severing* (*B* → *B* − 1, *P* → *P* − 1): Cofilin binding leads severing of a filament followed by its complete depolymerization.
- *Splitting* (*B* → *B* + 1, *P* → *P* + 1): Cofilin binding also under some conditions, leads to the further growth of the severed filament to an active filament.
- *Uncapping* (*P* → *P* + 1): The site where ARP2/3 is bound is also a capped pointed end. When it unbinds, an uncapped pointed end is formed, which is free to then depolymerize.

To indicate the state variables of different foci, we will use indexing in the following way: *B*^(*z*)^ is the number of barbed ends from focus *z*. Note, once the number of barbed ends reaches 0, we assume that there is no more filament to branch and the focus is removed from the simulation. Thus, it is also necessary that new foci are nucleated.

#### Nucleation

New actin foci within the spine are formed according to a nucleation rate *γ*_*nucl*_. The location for this – the nucleation points – are chosen probabilistically: first, a set of *n* points is generated which are at 80% of the distance between the membrane points and the origin. Second, the distance *d*_*j*_ of each of these points to the center of the PSD is calculated. Third, one of these points is selected as nucleation point with probability ${p}_{j}=\,{e}^{-\frac{{d}_{j}}{\lambda }}\left({\sum }_{l = 1}^{n}{e}^{-\frac{{d}_{l}}{\lambda }}\right)$, where *λ* is a scaling parameter for the PSD distance. The growth direction of the actin focus ${\overrightarrow{g}}^{(z)}$ is calculated as a unit vector along the line between origin and the nucleation point. If the membrane moves such that the nucleation point is outside the membrane, we move it backward along this direction vector until it is inside the spine again.

#### Actin force

We assume that each focus has a spatial extent orthogonal to its growth direction described by a Gaussian kernel with amplitude *α* and spread *σ* as

$$W(x)=\frac{\alpha }{\sigma \sqrt{2\pi }}{e}^{-\frac{{x}^{2}}{2{\sigma }^{2}}}$$

To calculate the force contribution of focus *z*, we first select all vertices ${\overrightarrow{x}}_{i}$ that lie in the growth direction from the focus origin. We then consider a line originating from the focus origin and extending in the growth direction ${\overrightarrow{g}}^{(z)}$ and calculate the orthogonal distance of these points ${o}_{i}^{(z)}$.

The actin force from focus *z* onto vertex *i* is then determined by this orthogonal distance and the barbed ends:

$${F}_{act,i}^{(z)}=W\left({o}_{i}^{(z)}\right){B}^{(z)}{\overrightarrow{g}}^{(z)}.$$

The contributions of each focus are summed at all vertices.

### Modeling LTP by time-dependent rates

The concentration of ABPs vary in a time-dependent manner in response to an LTP stimulus^15^. This time development can be fit with a double exponential function defined by two time constants - a rise time *τ*_1_ and a fall time *τ*_2_:

$$r(t)=A\left(1+B\frac{{e}^{-\frac{t}{{\tau }_{1}}}-{e}^{-\frac{t}{{\tau }_{2}}}}{{({\tau }_{1}/{\tau }_{2})}^{-1/({\tau }_{1}/{\tau }_{2}-1)}-{({\tau }_{1}/{\tau }_{2})}^{-1/(1-{\tau }_{2}/{\tau }_{1})}}\right)$$

Here, *A* is the basal rate and *B* is the percentage by which the rate changes. The values of these have been obtained from fitting measurements in^15^ and are reported in Table 1.

**Table 1:** Time-dependent model parameters varied through LTP

Process/protein	A	B	*τ*_1_(*s*)	*τ*_2_(*s*)	Comment/Source
Branching/ARP2/3	2.0	1.0	120	180	^22^ and ^15^
Capping/CapZ	1	0.3	120	360	^22^ and ^15^
Uncapping/ARP2/3	1/30	0.15	100	300	^22^ and ^15^
Severing/Cofilin	1	0.7	120	1680	^22^ and ^15^
Splitting/Cofilin	1	0.7	120	300	^15^
Nucleation	0.02	200	20	1680	adapted to experiment

### Model of the stable pool

We assume that filaments from dynamic pool transit to the stable pool through cross-linking with rate *k*_*b*_. Cross-linker unbinding leads to a shrinkage of the stable pool, and is proportional to its size and an unbinding rate *k*_*u*_. Thus we have

$$\frac{dS}{dt}={k}_{b}\underbrace{\mathop{\sum}_{z}{B}^{(z)}}_{{B}_{tot}}-{k}_{u}S$$

Therefore, our stable pool model behaves as an exponential low-pass filter of the total number of barbed ends with a timescale *k*_*u*_. Upon LTP-inducing stimulations, the cross-linker unbinding rate is instantly changed to *k*_*u*_ = 120 times the basal value and reset after 2 minutes.

The stable pool moreover also influences spine geometry. Assuming that it hinders retrograde movement, the expansive force from the dynamic actin filaments should proportionally grow with its size. We therefore replace the constant *α* in the kernel for our force equation by

$$\alpha (S)={\alpha }_{0}\left(1+q\,{f}_{S}\right) \,{{\mbox{with}}}\,{f}_{s}=\frac{S}{{B}_{tot}+S}.$$

Here *α*_0_ scales the stable pool independent force contributions and *q* the stable pool influence.

### Model parameters

We listed all time-constant parameters in Table 2. In comparison to^22,23^, we decreased the spine size to obtain realistic volumes and adapted a few parameters along that line.

**Table 2:** Time-independent model parameters

Name	Quantity	Value	Unit	Source/Comments
Cross-linker unbinding rate	*k*_*u*_	1/2820	*s*^−1^	Adapted to experiment
Cross-linker binding rate	*k*_*b*_	1/8037	*s*^−1^	Adapted to experiment
Proportionality constant	*ϕ*	75	*μm*^−1^	^22,23^
G-actin assembly rate constant	*k*_*on*_	11.6	*μM*^−1^*s*^−1^	^22,23^
Length of G-actin	*δ*	0.0022	*μm*	^22,23^
Thermal energy	*k*_*B*_*T*	0.0041	*pNμm*	^22,23^
Nucleation distance parameter	*λ*	0.1	*μm*	^22^ (Increased due to missing polarity of filaments ^52^)
Actin force amplitude (ctl)	*α*	0.5	*fN*	Adapted to experiment
Actin force amplitude	*α*_0_	0.5	*fN*	Adapted to experiment
Stable pool influence on force	*q*	10	-	Adapted to experiment
Spatial spread of each focus	*σ*	0.05	*μm*	^22^
Bending modulus	*κ*	0.18	*pNμm*	^22,23^
Elasticity of the membrane	*τ*	15	*pNμm*^−1^	^22,23^
Diff. between internal and external pressure	*p*	85.7143	*pNμm*^−2^	^22,23^
Speed of mesh movement	*ζ*	0.002	*μm*^2^*s*^−1^*pN*^−1^	^22,23^

#### Simulations

Simulations have been conducted and analyzed in python and numpy. With the selected time-step, the simulations can be executed approximately in real time on a consumer notebook. The simulation code can be found under^51^.

As a measure for the duration of the LTP induced changes we evaluate the duration until the difference between the current spine volume and its basal level has decayed to 10% of its maximal value.

### Abstract model for parameter fitting

In the following we will derive an analytical solution for the time-course of the stable pool and the necessary conditions for dynamic actin for matching the experimentally measured actin fraction.

We assume that, after cross-linkers start binding again, the dynamic pool *D* (which is proportional to the number of barbed ends) follows a time-course:

1 $$D(t)=\hat{D}{e}^{-{k}_{d}t}+{D}_{0}$$

while the stable pool follows

2 $$\frac{dS}{dt}=-{k}_{u}S+{k}_{b}D(t)$$

and is assumed to start at a value of *S*(0) = 0. This is an initial value problem based on a first order inhomogeneous linear ODE. The solution to the homogeneous equation (*D*(*t*) = 0) is

$${S}^{h}(t)={C}_{h}{e}^{-{k}_{u}t}$$

By variation of the constant *C*, we obtain a solution to the inhomogeneous case:

$$C(t)=\frac{{k}_{b}}{{k}_{u}-{k}_{d}}{e}^{({k}_{u}-{k}_{d})t}\hat{D}+\frac{{k}_{b}}{{k}_{u}}{e}^{{k}_{u}t}{D}_{0}$$

and arrive at

$$S(t)={C}_{h}{e}^{-{k}_{u}t}+\frac{{k}_{b}}{{k}_{u}-{k}_{d}}{e}^{-{k}_{d}t}\hat{D}+\frac{{k}_{b}}{{k}_{u}}{D}_{0}.$$

Using the initial value *S*(0) = 0, we obtain

$${C}_{h}=-{k}_{b}\left(\frac{\hat{D}}{{k}_{u}-{k}_{d}}+\frac{{D}_{0}}{{k}_{u}}\right)$$

Hence, the resulting time-course is a difference of exponentials.

3 $$S(t)=\frac{{k}_{b}}{{k}_{u}-{k}_{d}}\left({e}^{-{k}_{d}t}-{e}^{-{k}_{u}t}\right)\hat{D}+(1-{e}^{-{k}_{u}t})\frac{{k}_{b}}{{k}_{u}}{D}_{0}$$

4 $$f(t)=\frac{S(t)}{S(t)+D(t)}.$$

For the basal value at *t* → *∞*, this equates to

$$f(\infty )=\frac{\frac{{k}_{b}}{{k}_{u}}{D}_{0}}{\frac{{k}_{b}}{{k}_{u}}{D}_{0}+{D}_{0}}=\frac{{k}_{b}}{{k}_{b}+{k}_{u}}$$

Thus, for a given (or measured) basal value of *f*(*∞*) = *f*_*b*_, we can constrain the ratio between binding and unbinding rate of the cross-linkers:

$${f}_{b}=\frac{{k}_{b}}{{k}_{b}+{k}_{u}}\Rightarrow {k}_{b}=\frac{{f}_{b}}{1-{f}_{b}}{k}_{u}=:{F}_{b}{k}_{u}.$$

Using this relation in the time-course results in

5 $$S(t)={F}_{b}\frac{{k}_{u}}{{k}_{u}-{k}_{d}}\hat{D}\left({e}^{-{k}_{d}t}-{e}^{-{k}_{u}t}\right)+{F}_{b}{D}_{0}(1-{e}^{-{k}_{u}t}).$$

### Parameter fitting

We compare the time courses obtained from equations (1), (5) and (4) to the fractions of stable actin measured in the FRAP experiments. The actual time point when LTP started is not completely determined, as the cLTP protocol is applied for 15 minutes. Hence, we add a time shift Δ*t* to the measurement times (30, 90 and 150 minutes), which is bounded between 0 and 15 minutes, and evaluate the model at these times. We then determine the mean squared error between the model and the measured stable actin fractions, weighted by the inverse of the squared error of the measurement errors (in this case the standard deviations from the stable fraction fits). We then iterate through different combinations of timescales *τ*_*u*_: = 1/*k*_*u*_ and *τ*_*d*_: = 1/*k*_*d*_, and optimize the initial dynamic pool value $\hat{D}$, the time shift Δ*t*, and the basal actin fraction *f*_*b*_. We limit the possible ranges to $\hat{D}\in [0,200\cdot {D}_{0}]$, $\Delta t\in [0,15\min ]$ and $f_{b} \in [0, 0.4]$ and then minimize these parameters with respect to the weighted mean squared error using the scipy’s minimize function and the Powell method.

We then transfer the best fitting parameters to our model with *D* and *τ*_*d*_ being the amplitude and decay timescale of the LTP induced change in the focus nucleation rate, as well as *F*_*b*_*k*_*u*_ and *k*_*u*_ being the binding and unbinding rate for the stable pool. With this, we finally adjusted the force parameters *α*_0_ and *q* in order to obtain spine volume time courses comparable to experiments^19^—that is with a fast rise (adjusted by *q*) and maximal volume change of around 150–200% (see Supp. Figs. 3 and 4), which matches with the initial time-course of spine volumes after glutamate-uncaging induced LTP^19^.

### Statistics and reproducibility

For each simulated condition (control, without and with stable pool as well as parameter tests), we conduct *n* = 20 simulations and depict mean and standard deviation values.

FRAP analyses were conducted for *n* = 16, 12, 9 and 11 for the control condition as well as 30, 90 and 150 minutes after cLTP, respectively, with spines stemming from two independent cultures for each condition. Statistical differences were evaluated for the means over the last 36 acquired time-points using a Kruskal Wallis and post-hoc Dunn test.

### Reporting summary

Further information on research design is available in the Nature Portfolio Reporting Summary linked to this article.

## Supplementary information

Supplemental Information

Reporting Summary

## Data Availability

Simulation and experimental results for the reproduction of the above analyses are available under 10.25625/TSDKO3^51^. All other data are available from the corresponding author (or other sources, as applicable) on reasonable request.

## Code availability

Simulation code for reproduction of the above results is available under 10.25625/TSDKO3^51^.
