# Supplementary material for: Long-term potentiation-induced changes in actin dynamics and spine geometry persist on the timescale of the synaptic tag
Source: Commun Biol. 2025 Jul 18;8:1065. doi: 10.1038/s42003-025-08459-0 (PMC12274606; doi:10.1038/s42003-025-08459-0)
Supplement: Supplementary file 1 — Supplemental Information [file 42003_2025_8459_MOESM1_ESM.pdf]

Supplementary Information for: Long-term potentiation-induced changes in actin dynamics and spine geometry persist on the timescale of the synaptic tag.

Mitha Thomas, Cristian-Alexandru Bogaciu, Silvio O. Rizzoli, Michael Fauth

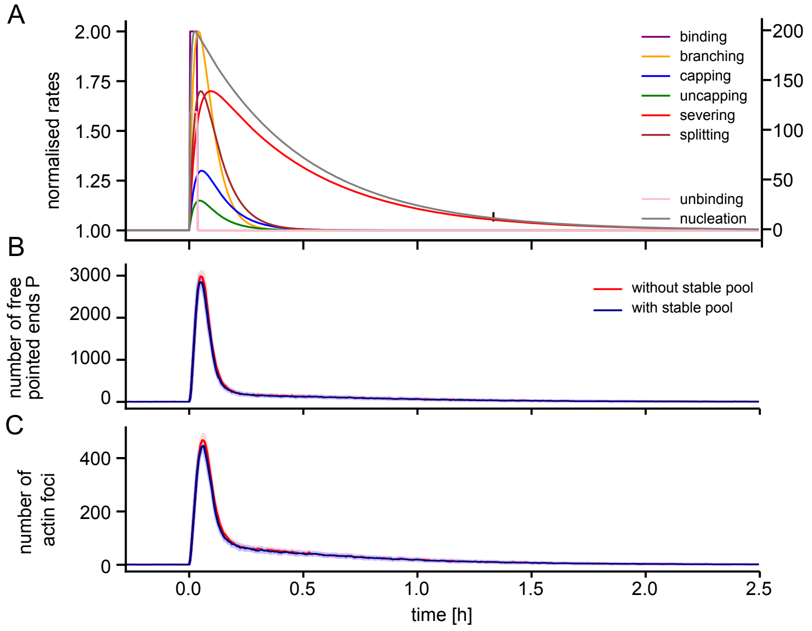

Suppl. Fig. S1 Time-courses of further model quantities: (A) Time-course of normalized rates in simulation (right axis applies for nucleation and unbinding). (B) Time-course of the number of uncapped pointed ends P. Curves show mean and standard deviations over 20 simulations (C) Same for number of polymerization foci n_f.

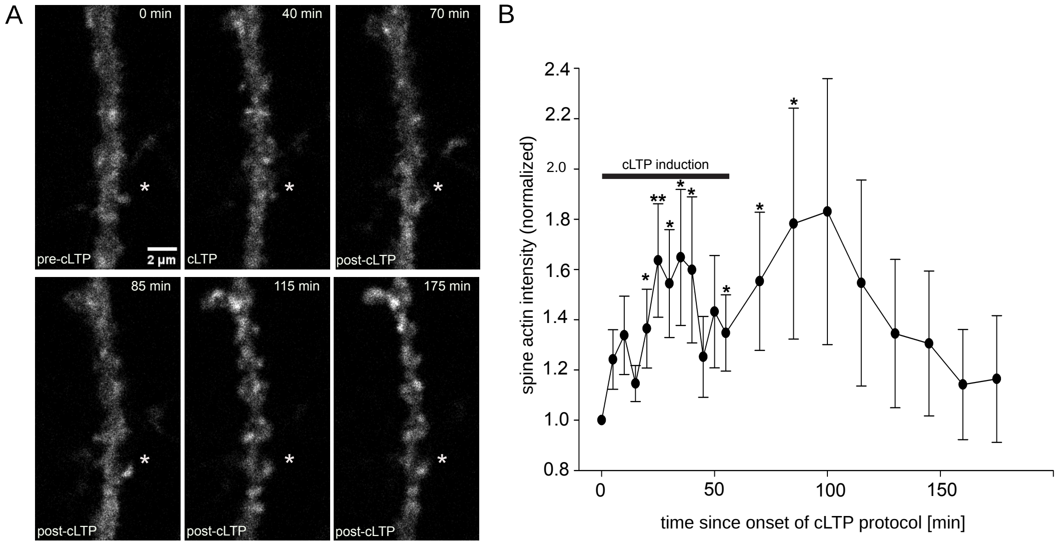

Suppl. Fig. S2 Spine modifications induced by cLTP. (A) Typical images showing actin in spines of hippocampal cultured neurons before, during and after cLTP induction. One exemplary spine is indicated. Scale bar 2 µm. (B) Quantification of actin intensity in spines before, during and after cLTP. 62 spines, from 2 independent experiments, were analysed. All traces were normalized to the initial, pre-cLTP, starting point. Means ± SEM of the normalized intensities from all technical replicates (62 spines) are shown. During cLTP induction, the actin intensity is significantly higher than the initial baseline ($*p = 0.032$, 0.028, 0.038 and 0.044 for the fifth, seventh, eighth and ninth indicated time points and $**p = 0.004$ for the sixth time point). At the end of the cLTP-inducing stimulus, the actin intensity is significantly higher than the initial baseline ($*p = 0.0216$, 0.0166 and 0.0491 for the three indicated time points), but it decays afterwards, reaching values close to the baseline at two hours after the termination of the cLTP-inducing stimulus (Kruskal-Wallis test, with overall p=$0.44 \cdot 10^{-6}$, followed by Fisher's post hoc test). Source data can be obtained from [1].

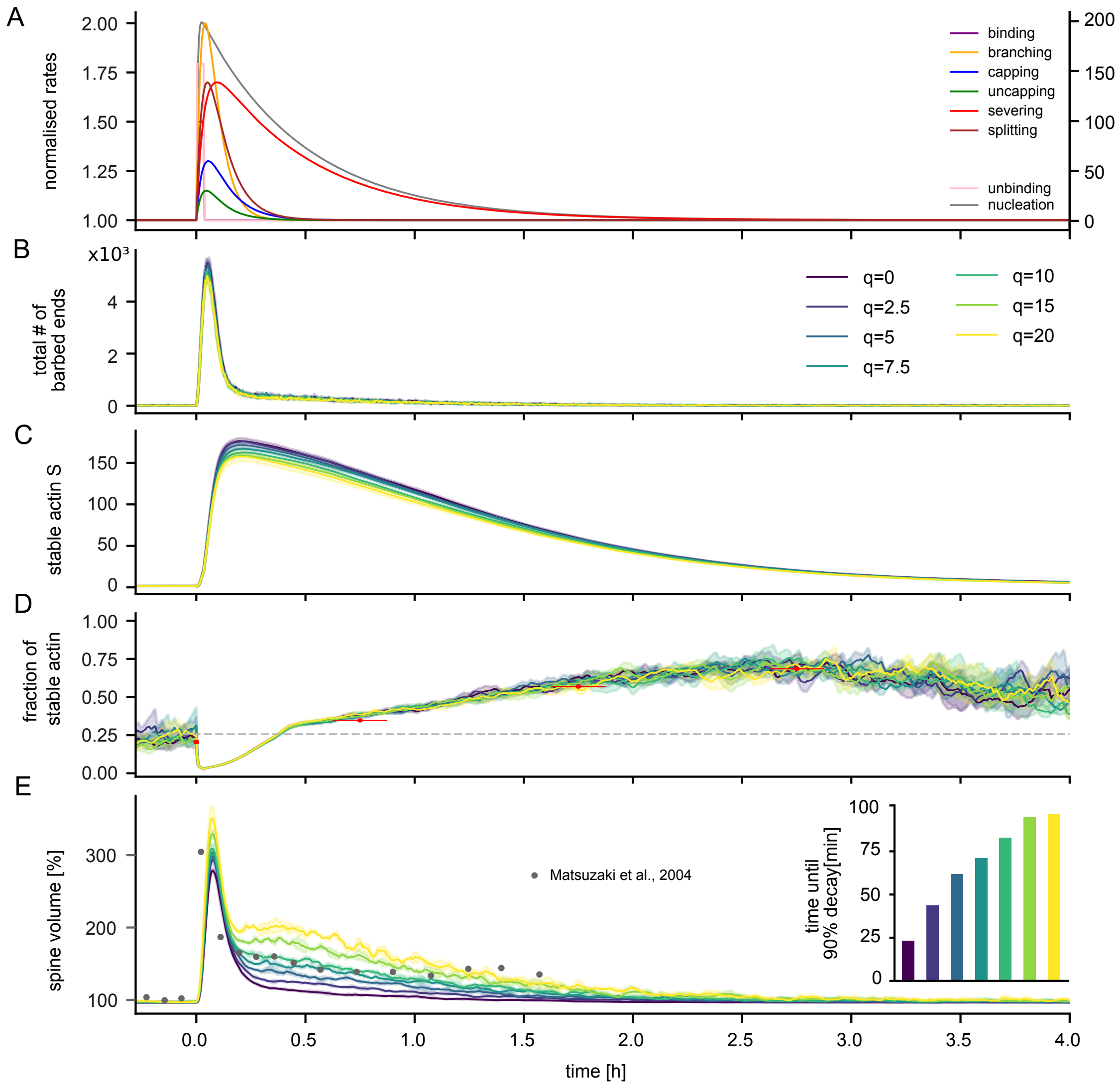

Suppl. Fig. S3 Variation of stable pool scaling parameter q (A) Time-course of normalized rates determining actin dynamics (right axis applies for nucleation and unbinding only). (B) Total number of barbed ends in the spine (summed over all polymerization foci). Colors indicated different values of q as indicated. Curves depict mean (solid) and standard deviation (shaded) over 3 simulations of the stochastic actin dynamics. (C) Time-course of the stable pool. (D) Time-course of the fraction of actin allocated to stable pool. Red crosses signify the measured data points from Fig. 2. (E) Time-course of the volume of the spine. Grey dots mark experimentally obtained spine volumes from L-LTP [2] to which we compare to arrive at plausible values of q and α_0. Best matching value was $q = 10$. (Inset) Time until 90% of the volume increase have decayed for models with and without stable pool.

Whereas the number of barbed ends and the stable pool dynamic remain unaltered by q, it alters the time-course of the spine volume. Larger values prolong the time interval in which the volume decays back to its basal value.

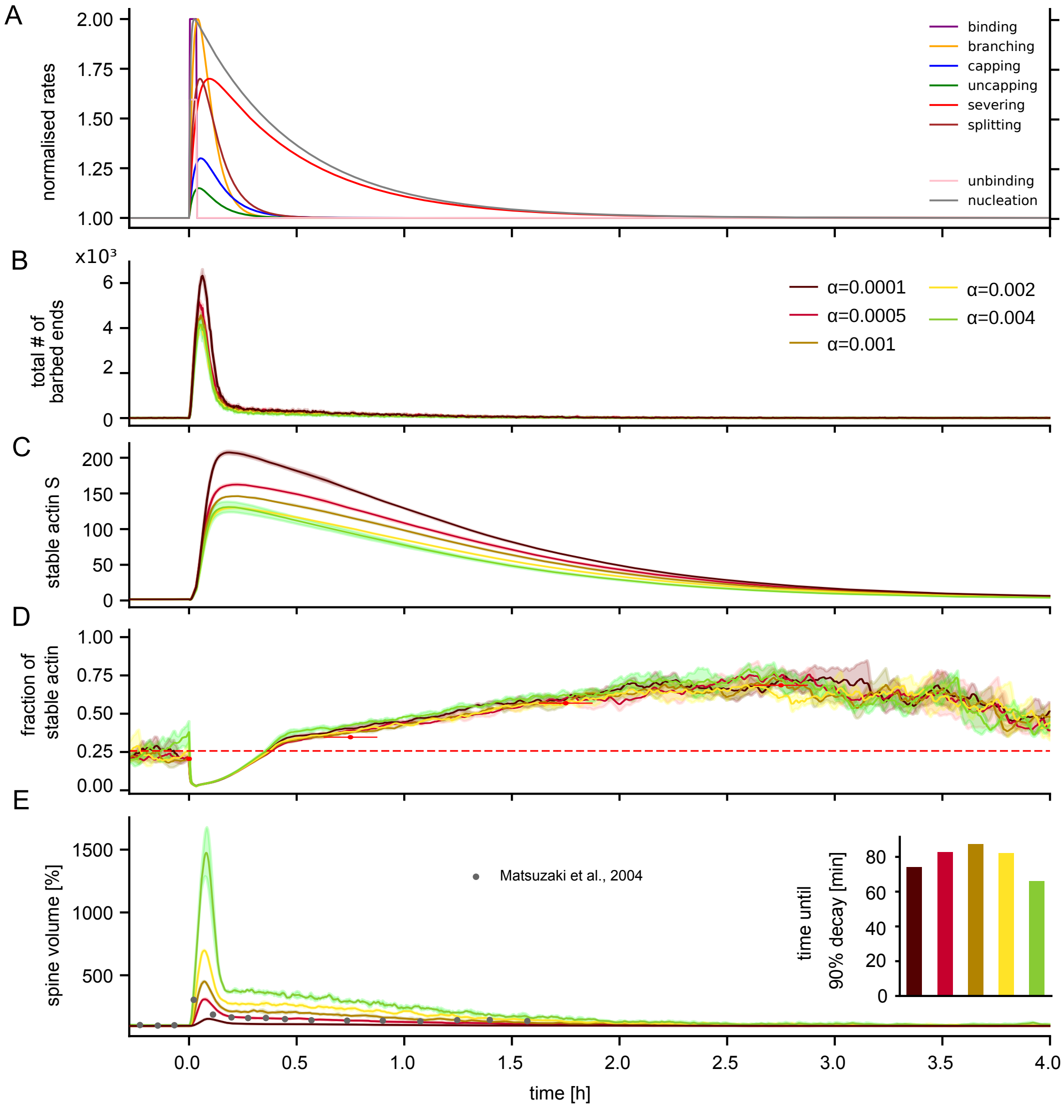

Suppl. Fig. S4 Variation of force magnitude α_0 (A) Time-course of normalized rates determining actin dynamics (right axis applies for nucleation and unbinding only). (B) Total number of barbed ends in the spine (summed over all polymerization foci). Colors signify the value of α_0 as indicated. Curves depict mean (solid) and standard deviation (shaded) over 3 simulations of the stochastic actin dynamics. (C) Time-course of the stable pool. (D) Time-course of the fraction of actin allocated to stable pool. Red crosses signify the measured data points from Fig. 2. (E) Time-course of the volume of the spine. Grey dots mark experimentally obtained spine volumes from L-LTP [2] to which we compare to arrive at plausible values of q and α_0. Best matching amplitude was $\alpha_0 = 0.00005$ pN. (Inset) Time until 90% of the volume increase have decayed for models with and without stable pool.

Large values of α will lead to large forces and membrane counter-forces. Thus, polymerization is hindered and dynamic and stable actin pools are attenuated (whereas their fraction remains approximately the same). The value of α scales the time-course of the volume while leaving its shape approximately the same. Thus, there are no large differences in the time until 90% of the LTP-induced changes have decayed.

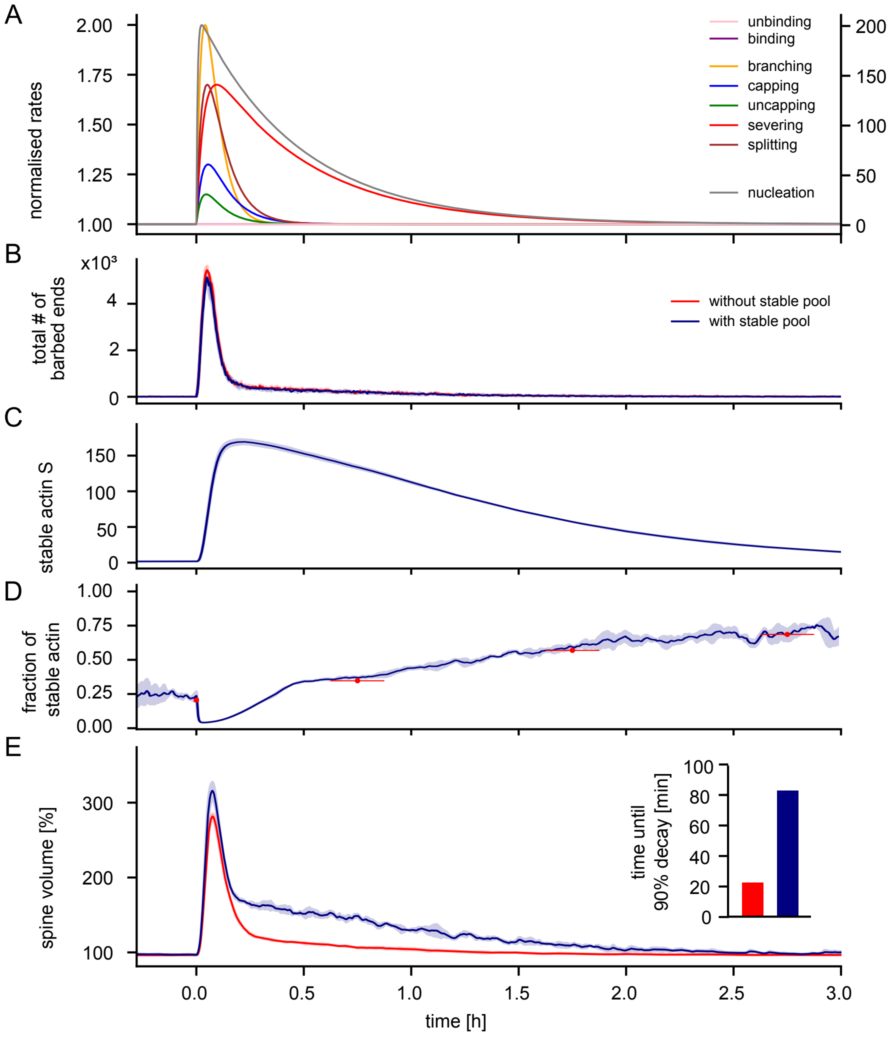

Suppl. Fig. S5 Simulations without cross-linker alteration (A) Time-course of normalized rates determining actin dynamics (right axis applies for nucleation only). (B) Total number of barbed ends in the spine (summed over all polymerization foci). Blue curves show simulations using the stable pool and the altered ABP-rates from panel A, and red curves the model without the stable pool (see Fig. 1). Curves depict mean (solid) and standard deviation (shaded) over 20 simulations of the stochastic actin dynamics. (C) Time-course of the stable pool. (D) Time-course of the fraction of actin allocated to stable pool. Red crosses signify the measured data points from Fig. 2. (E) Time-course of the volume of the spine. (Inset) Time until 90% of the volume increase have decayed for models with and without stable pool.

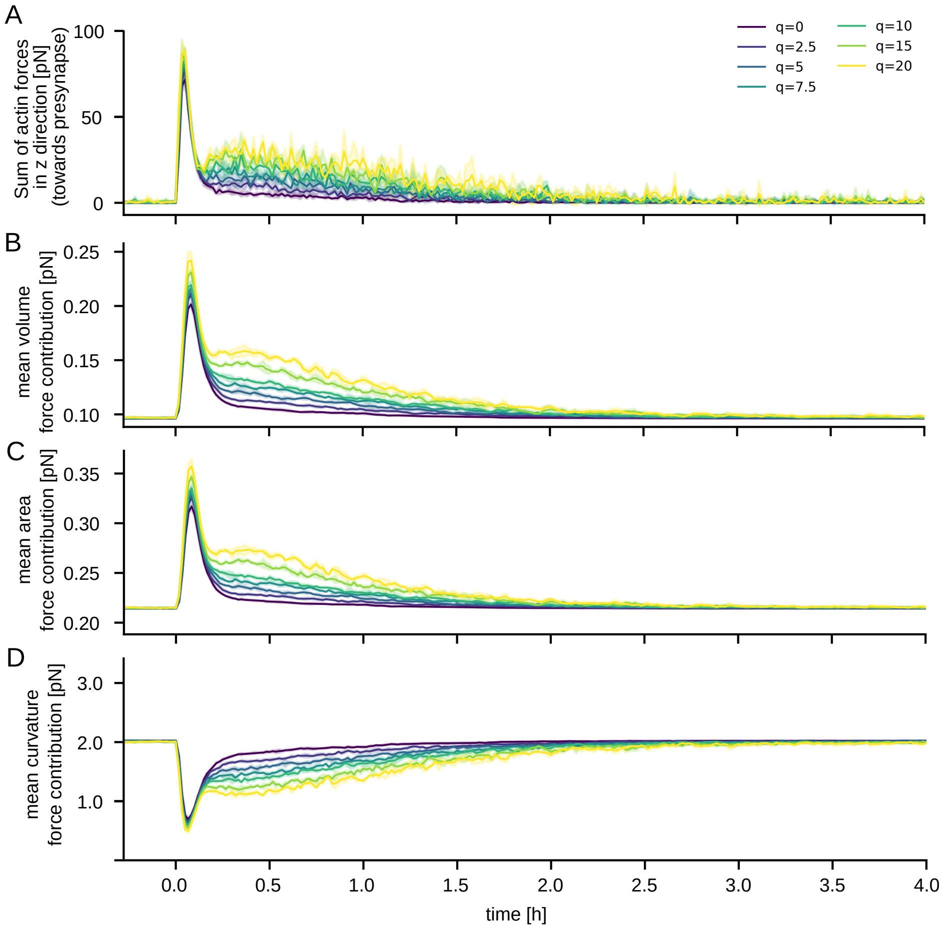

Suppl. Fig. S6 Forces in the model: (A) Time-course of the sum of actin forces in z-direction (towards presynapse) over the upper half of the spine. Colors indicate different values of scaling parameter q. (B) Time-course of mean magnitude of the volume-contribution of the membrane force for the upper half of the spine. (C) Same for the area contribution (D) Same for the curvature contribution. The major force contribution stems from curvature.

References

[1] Thomas, M., Bogaciu, C.-A., Rizzoli, S.O., Fauth, M.: Replication Data For: LTP-induced Changes in Actin Dynamics and Spine Geometry Persisting on the Timescale of the Synaptic Tag. https://doi.org/10.25625/TSDKO3

[2] Matsuzaki, M., Honkura, N., Ellis-Davies, G.C., Kasai, H.: Structural basis of long-term potentiation in single dendritic spines. Nature **429**(6993), 761–766 (2004)
